# Prevention of Post‐Inflammatory Hyperpigmentation in Skin of Colour: A Systematic Review

**DOI:** 10.1111/ajd.14432

**Published:** 2025-02-14

**Authors:** Kristie Mar, Mahan Maazi, Bushra Khalid, Rayan Ahmed, Ou Jia (Emilie) Wang, Touraj Khosravi‐Hafshejani

**Affiliations:** ^1^ Faculty of Medicine University of British Columbia Vancouver British Columbia Canada; ^2^ Department of Dermatology and Skin Science University of British Columbia Vancouver British Columbia Canada

**Keywords:** FST, laser, pigment, PIH, sunscreen

## Abstract

**Background/Objectives:**

Post‐inflammatory hyperpigmentation (PIH) impacts all skin tones with a heightened predilection for Fitzpatrick skin types (FST) III‐VI. Preventative measures include pre‐ and post‐intervention approaches, such as sunscreen and corticosteroids. This systematic review aims to summarise the preventative measure outcomes for skin of colour individuals.

**Methods:**

A literature search was conducted using MEDLINE (from 1946) and Embase (from 1974) in adherence to the Preferred Reporting Items for Systematic Reviews and Meta‐Analysis guidelines.

**Results:**

Of 14 studies, 369 cases were included. The mean age was 38 years (*n* = 293) and 72% were female (*n* = 265). All patients were of Asian ethnicity, and 42% were of FST III, 54% FST IV, and 4% FST V. Nearly all cases were precipitated by laser therapy (> 95%), and the face was the most reported location (85%). The most successful preventative measure was sunscreen alone or combined with other ingredients. Less successful outcomes were seen with topical corticosteroids and systemic tranexamic acid, while cooling air devices exacerbated the development of PIH.

**Conclusion:**

Overall, only sunscreen consistently prevented the incidence of PIH; however, the severity of the ensuing PIH may be diminished with other measures. There is considerable room for improved preventative strategies for at‐risk populations.

## 1 Introduction

Post‐inflammatory hyperpigmentation (PIH) is a form of acquired hypermelanosis often observed as a consequence of endogenous factors, such as inflammatory conditions, or exogenous factors, such as mechanical trauma [1, 2, 3, 4]. This condition is characterised by an excess production and/or deposition of melanin in the epidermis and/or dermis, resulting from inflammation [1, 2, 3]. PIH tends to be more prominent and enduring in individuals with darker skin tones, particularly in Asian and darker‐skinned populations (Fitzpatrick Skin Type [FST] III‐IV) individuals [5]. This is due to an increased size of melanosomes and quantity of melanin, as well as increased eumelanin [3, 6].

Women with skin of colour (SOC) exhibit a particularly high concern for clearing PIH [7]. Despite its significant impact, research on PIH has traditionally centred around individuals with lighter skin tones, leading to noticeable disparities in care [8]. Preventative approaches investigated in the general population include topical retinoids, hydroquinone, corticosteroids and sunscreen [7, 9]. Of interest, recent studies have begun to specifically examine prevention for SOC patients, given the reported incidence range of 10%–17% when undergoing dermatologic procedures [7, 10, 11, 12, 13, 14, 15, 16, 17].

Reviews in 2014, 2017 and 2020 summarised treatments and preventative measures in the general population [9, 16, 17]. In 2012, a literature review was conducted on acne‐induced aesthetic procedure‐related PIH, particularly for Asian patients [18]. However, an updated review on preventative outcomes for PIH is currently lacking for this at‐risk population. The overarching goal of this review is to summarise the available literature for the prevention of PIH specifically for SOC and equip physicians with knowledge to improve patient outcomes for a diverse range of skin tones.

## 2 Methods

This systematic review was conducted with adherence to the Preferred Reporting Items for Systematic Reviews and Meta‐Analyses (PRISMA). The study protocol was registered with the PROSPERO database (CRD42022366067).

### 2.1 Eligibility Criteria

Eligibility criteria for this review were established as follows:

- Population: Individuals of any age and sex with SOC (FST III‐VI, non‐Caucasian ethnicity/race) undergoing preventative measures for PIH, regardless of the underlying condition or comorbid conditions.
- Intervention: Any preventative intervention aimed at addressing PIH was considered.
- Comparator: The review compared SOC individuals who received prevention versus those without prevention.
- Outcomes: Presence or absence of PIH, scoring indexes for PIH (e.g., IGA, PGA), follow‐up duration, adverse effects.
- Study Design: Eligible studies included randomised controlled trials, observational research such as cohort (prospective and retrospective), cross‐sectional, case–control studies, case‐series and case reports.

### 2.2 Search Strategy

A literature search was conducted using MEDLINE (from 1946), Embase (from 1974) and PubMed on July 2, 2023, using the OVID interface. Additionally, Cochrane Database of Systematic Reviews was searched, and reference lists of included studies were reviewed. The MeSH term ‘postinflammatory hyerpigmention’ and keywords ‘post‐inflammatory hyperpigmentation, hypermelanosis, hyperchromia, dyschromia’ were used. Date, geographical, or language restrictions were not applied. Title, abstract and full‐text screening was conducted independently by three reviewers (K.M., B.K., and M.M.) using Covidence online systematic review software (www.covidence.org). During the full‐text screening stage, studies were excluded if they did not follow each component of the predetermined PICOS criteria. Any conflicts were resolved through consensus.

### 2.3 Data Extraction

Data extraction was completed by three reviewers (K.M., B.K. and M.M.) on a standardised extraction form. We extracted data on the publication year, country, study design, number of participants, age and sex of participants, race/ethnicity, FST, preventative regimen, occurrence of PIH, scoring indexes, follow‐up duration and adverse effects.

### 2.4 Data Synthesis

The lack of standardised reporting necessitated a pooled and separate reporting of results. For studies with certain variables, such as scoring indexes, outcome significance, and patient satisfaction that could not be pooled, a narrative synthesis was completed. For pooled intervention results, outcomes were reported as an absence or presence of PIH.

### 2.5 Definitions

- Absence of PIH: Successful prevention of PIH, with no detectable PIH, as defined by the criteria outlined in each study. For example, one study used the Brown score and found ‘no significant changes from baseline to six weeks’.
- Presence of PIH: Failure to prevent PIH, with detectable PIH, defined by the criteria outlined in each study. For example, one study used the Hyperpigmentation area and severity index (HASI) and found ‘significant difference … at both the eight‐ and 12‐week follow‐up’.

## 3 Results

### 3.1 Patient Characteristics

**FIGURE 1:**
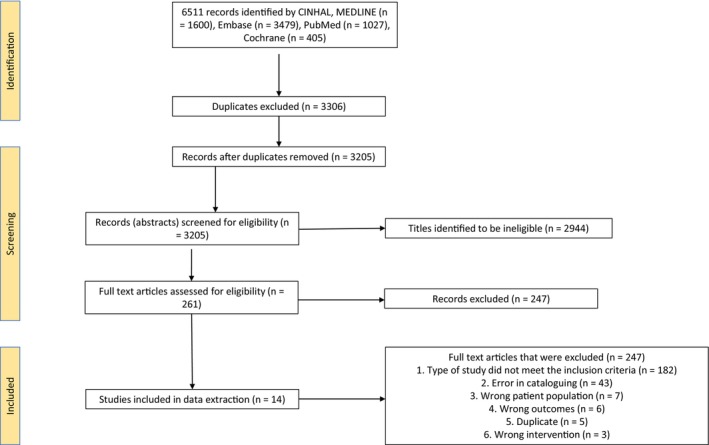
PRISMA flow diagram.

After title and abstract screening of 3205 articles and full‐text review of 261 articles, 14 studies were ultimately included in this review, summarising 369 patients (Figures 1 and S1). Most of the studies came from Thailand (57%), Korea (14%), the United States (7%), China (7%), and Italy (7%) (Table S1). Randomised controlled trials (57%), experimental studies (21%), and case reports (14%) were the most frequent study types.

The mean age was 38 years (*n* = 293) and 72% were female (*n* = 265) (Table 1). Studies that investigated the prevention of PIH reported that 100% were Asian (*n* = 178). Among 339 cases, Fitzpatrick skin type III accounted for 42%, type IV for 54%, and type V for 4%.

**TABLE 1:** SOC patient characteristics and clinical features of PIH.

	SOC Patients
Total number of patients (*n*)	369
Sex (%)	
Females	71.8
Males	28.2
Total	369
Pooled mean age (years)	37.9
Total	293
Ethnicity/Race (%)	
Black	0
Asian	100.0
Hispanic/Latin	0
Other	0
Total	178
Fitzpatrick skin type (%)	
I	0
II	0
III	42.2
IV	53.7
V	4.1
VI	0
Total	339
Location (%)	
Face	85.3
Extremities	9.0
Trunk	5.8
Total	278
Precipitating factor (%)	
Trauma	
Laser therapy	95.4
Chemical peel	0.3
Ultraviolet‐B irradiation	4.3
Total	369

### 3.2 Precipitating Factors

Studies that investigated the prevention of PIH were only cases of PIH exacerbated by trauma (Table 1). Among 369 cases, 95% of the cases (*n* = 352) were caused by laser therapy, 4% (*n* = 16) from ultraviolet‐B (UVB) irradiation and 0.3% caused by chemical peels (*n* = 1). The most common laser identified was fractional carbon dioxide (CO_2_) (43%), followed by 532‐nm Q‐switched (QS) neodymium‐doped (Nd): yttrium aluminium garnet (YAG) (25%), picosecond (17%) and 1064‐nm QS Nd:YAG (15%). The face was most heavily affected (85%), followed by extremities (9%) and trunk (6%). Both the experimental and control groups experienced identical precipitating factors for their PIH in each study.

### 3.3 Interventions and Outcomes

#### 3.3.1 No Intervention

Among the control group of 74 (18%), who only received a regular skin care routine, 34% achieved successful prevention of PIH, while 66% experienced a failure in prevention (Table 2). Participants were followed for a mean duration of two months (*n* = 55).

**TABLE 2:** Preventions and associated outcomes for SOC patients.

Prevention	Sample (%)	Mean number of treatments	Absence of PIH (%)	Presence of PIH (%)	Follow‐up (months)
No prevention	15.8	35.6	33.8 (25/74)	66.2 (49/74)	2.5
Total	74				55
*Monotherapeutic topical regimen (51.2%)*					
Sunscreen	13.2	21.0	98.4 (61/62)	1.6 (1/62)	1.5
Total	62				62
Topical antibiotic	12.6	168.0	0 (0/59)	100.0 (59/59)	4.0
Total	59				59
Topical EGF	13.2	63.0	33.9 (21/62)	66.1 (41/62)	1.7
Total	62				43
Topical steroid	12.2	4.0	58.5 (24/41)	41.5 (17/41)	3.1
Total	57				41
*Combination topical regimen (18.4%)*					
Sunscreen, anti‐inflammatory, other	18.2	31.3	100.0 (85/85)	0 (0/85)	2.0
Total	85				85
Topical brimonidine, methylprednisolone	0.2	3.0	100.0 (1/1)	0 (0/1)	6.0
Total	1				1
*Combination topical/systemic regimen (0.2%)*					
Sunscreen, topical retinoid, oral TXA, other	0.2	—	100.0 (1/1)	0 (0/1)	—
Total	1				
*Monotherapeutic systemic regimen (9.6%)*					
Systemic TXA	9.6	—	0 (0/43)	100 (43/43)	3
Total	45				20
*Devices (4.5%)*					
Cooling air device	4.5	40.0	0 (0/21)	100.0 (21/21)	—
Total	21				

Abbreviations: EGF, epidermal growth factor; TXA, tranexamic acid.

#### 3.3.2 Sunscreen

The most common preventative measure investigated was sunscreen incorporated into different regimens (31%, *n* = 148). Sunscreen alone or in combination with anti‐inflammatory ingredients exhibited preventative capability, and no specific regimen showed superiority over the other. Available data from two studies revealed 85 (18%) participants trialled sunscreen combined with licochalcone A, L‐carnitine, and avobenzone, which are anti‐inflammatories and antioxidants. Patients applied sunscreen daily for a mean of 31 days following picosecond or fractional CO_2_ laser skin resurfacing. A 100% success rate in prevention of PIH was reported with a follow‐up period of two months (*n* = 85). One study used the Brown score and found no significant changes from baseline to six weeks in 59 patients [19]. The second study could not prevent incidence in all 26 patients but reduced the onset and duration of the transient PIH that did occur [20]. The control group exhibited a 4% increase in melanin index (MI) from baseline, while the experimental group exhibited a 0.5% increase after one week. Of note, there was no persisting PIH or significant difference in MI by the end of the four weeks in either group.

Sunscreen alone was used by 62 participants (13%) with a mean of 21 applications following one session of laser treatment. All participants but one (98%) successfully prevented PIH, with a follow‐up of two months (*n* = 62). In one study, after participants received a session of picosecond laser, investigators reported non‐significant differences in the Brown score between baseline and six weeks [19]. Moreover, when compared to sunscreen with anti‐inflammatories, no significant difference was found [19].

#### 3.3.3 Epidermal Growth Factor

Topical endothelial growth factor (EGF), used by 62 (13%) participants, applied 63 times, achieved a 34% success rate in prevention but failed to prevent in 66% of cases. Of the total participants, 43 of them were followed up within two months. Available data revealed 19 participants received a single treatment of fractional CO_2_ [21], 13 received 532‐nm QS Nd:YAG [22], and 30 received 1064‐nm QS Nd:YAG [23], followed by daily applications of EGF. All three studies reported an improvement in the appearance of PIH, but the incidence of PIH between treatment and control groups was insignificant. However, patient satisfaction in the treatment group was significantly higher [23].

#### 3.3.4 Tranexamic Acid

Systemic tranexamic acid (TXA), used by 45 (10%) individuals, failed to prevent PIH from occurring in all, with a mean follow‐up time of 12 weeks (*n* = 20). One study found equivocal efficacy with TXA 1500 mg/day P.O. following 532‐nm QS Nd:YAG laser treatment in 20 individuals [24]. At six weeks, results did not differ through clinical examination between the treatment and control groups, yet dermoscopic examination showed significantly increased clearance of PIH at six and 12 weeks post‐treatment. Moreover, intradermal TXA (50 mg/mL) failed to prevent the incidence of PIH after treatment with a 532‐nm QS Nd:YAG laser in 25 patients [25]. There were no significant differences in the mean MIs between the treatment group and control group by the end of the study, nor was there a significant difference in relation to clearance of the pigmentation. However, a case report observed successful prevention in a patient treated with a combination of sunscreen, tretinoin 0.05%, spironolactone 100 mg, and oral TXA 650 mg daily after chemical peels containing lactic and salicylic acid [26]. No new PIH was observed throughout nine peels over the span of 2.5 years.

#### 3.3.5 Topical Antibiotics

Topical antibiotics utilised by 59 (13%) participants, applied an average of 168 times, failed to prevent PIH in all. Participants were followed up for a mean of four months. Fusidic acid cream failed to prevent PIH after fractional CO_2_ laser treatment, but decreased PIH severity, when applied twice daily for seven days in 30 individuals [27]. A significant difference in the HASI was evident between groups: the fusidic acid group exhibited a 2% and 0.7% increase in HASI score at eight and 12 weeks, respectively; in contrast, the erythromycin group demonstrated a 5% and 3% increase in HASI score.

#### 3.3.6 Topical Corticosteroids

Topical corticosteroids alone were utilised in 57 (12%) patients, with an average of four applications and a follow‐up of three months. Of those, 58% prevented PIH and 42% failed to prevent PIH. One study with 40 patients using topical clobetasol after a single fractional CO_2_ laser session failed to prevent PIH [28]. However, it significantly decreased its intensity and size [28]. Of note, three patients (8%) developed acneiform eruptions on the third and fourth days postoperatively. One individual undergoing 532 QS Nd:YAG laser treatment applied topical steroids alone and had an occurrence of PIH observed on dermoscopy after the procedure [29]. The third study reported that UVB irradiation‐induced PIH improved in 16 patients using either 0.05% clobetasol or 0.1% hydrocortisone butyrate cream, although no definitive incidence rates were provided [30]. Topical steroids reduced the MI significantly at 24 h and seven days post‐treatment. This effect was a more pronounced reduction with clobetasol compared to the hydrocortisone.

A single participant (0.2%) underwent 532 nm QS Nd:YAG laser treatment [29] and had methylprednisolone applied for three days and brimonidine gel for three weeks [29]. Dermoscopic examination showed no evidence of PIH in the treated lesion. No adverse events were reported after a follow‐up at six months.

#### 3.3.7 Devices

Cooling air devices, utilised by 21 participants (5%) after a session of 1064‐nm QS Nd:YAG laser, failed to prevent PIH in all, with no healing or follow‐up data reported. Patients received cooling to half the face during and 30 s before and after laser, set at a cooling level of four. Not only did it fail to prevent, but there was a relative risk of 2.6 with cooling. Additionally, one patient with FST IV developed persistent PIH on the cooled side.

## 4 Discussion

PIH poses challenges, particularly for SOC individuals receiving dermatologic treatment for underlying conditions, and its prevention can be challenging. Among the available studies, only sunscreen demonstrated consistent preventative capability to decrease the incidence of clinically evident PIH. However, the severity of the ensuing PIH may be diminished with other preventative measures.

Our study observed a higher prevalence of females in prevention studies who received targeted intervention for their face. Perhaps the intersection of being female and having skin of colour may compound the impact on QoL and heighten interest in seeking treatment [31, 32, 33]. However, it is crucial to emphasise that PIH occurs from other endogenous and exogenous causes; it affects all skin tones and other bodily locations as well.

Historically, dermatologic research has lacked documentation of race/ethnicity [8, 34]. Much of the earlier research focused on Caucasian individuals, inferred from recorded FST, or included images [8, 34]. Our study initially aimed to capture all SOC; however, the preventative measure of PIH has solely been studied on Asian patients. The predominant FST was type IV, followed by type III. Currently, the literature lacks any individuals with FST type VI. Therefore, it is imperative to expand research inclusivity to encompass a diverse range of ethnicities.

Dark‐skinned individuals exhibit a heightened predisposition to PIH, attributed to molecular, cellular, and structural factors [35]. Skin pigmentation level has been linked to melanocyte size, with larger cells in dark skin transferring more melanosomes to the epidermis due to heightened tyrosinase activity and larger, more acidic dendrites compared to light skin [4, 36, 37]. Investigators have also found increased oxidative stress, impaired cutaneous vascular function, and elevated inflammatory markers like interleukin‐6 and C‐reactive protein in African Americans [4]. Additionally, structural differences in the epidermis and dermal‐epidermal junctions enhance the susceptibility to PIH [4]. Furthermore, dark‐skinned individuals are less likely to apply photoprotection [4].

Our study found that trauma, primarily through fractional CO_2_ laser or 532‐nm QS Nd:YAG laser, was the precipitating factor in studies that investigated methods of PIH prevention. Prior literature reported an incidence rate spanning 10%–20% among SOC undergoing laser therapy [15, 35]. The exact mechanism is unclear but likely involves heat production [10]. Proper delivery of laser energy can mitigate but not fully eliminate the risk of PIH [10]. Longer wavelengths, such as 1064 nm, are considered safer for darker skin tones due to efficient absorption of energy by dark skin, necessitating a lower energy input to attain comparable outcomes with lighter skin [10], and deeper penetration to target dermal melanin with relative sparing of the epidermis [38]. Picosecond lasers, known for emitting short pulses, are also recognised as safe for darker skin tones due to their ability to minimise unwanted heat damage [10, 39].

Without prevention, our analysis revealed that most individuals developed PIH. The only interventions with lower incidence rates were sunscreen and topical steroids. The effectiveness of other interventions raises the question of whether they should be used at all. It is frequently noted that PIH that does develop tends to be transient, and over time, there is often little disparity between treatment and control groups. However, there exists a subtlety in the severity of the PIH that does manifest, which poses challenges in quantification. Additionally, there is a recognised possibility that developed PIH may persist for an extended duration or even indefinitely [5]. Consequently, one could argue convincingly for the benefits of prevention.

Either alone or in formulations containing anti‐inflammatories, sunscreen successfully prevented PIH for both acne and laser‐induced PIH, aligning with existing literature [10, 40]. Photoprotection is standard post‐laser care for all skin tones and should be practiced for at least two weeks before a procedure for optimal results [9]. However, it is generally neglected for SOC individuals in other circumstances. For example, surveys in the United States have shed light on societal trends, showing that photoprotection ranks lower in recommendations for dyschromia in African Americans compared to White populations [40]. This underscores the importance of inclusive education [10, 40].

EGF, TXA and fusidic acid have shown limited efficacy in addressing PIH. Topical EGF use was based on studies revealing associations between EGF and increased melanocyte stimulation [40]. However, Ying et al.'s meta‐analysis revealed a very limited transient inhibitory effect of topical products containing EGF on PIH in the general population [41]. Taken together, this suggests that EGF may not serve as a successful preventative agent for anyone.

While the mechanism of TXA remains unclear, it is believed to block melanocyte‐keratinocyte interactions, disrupting the inflammatory process [24, 26]. Currently, the sample size limits any conclusive findings regarding various routes of administration; however, it may play a role as a complementary agent in PIH clearance, and further research is warranted.

Similarly, fusidic acid, proposed to reduce laser‐induced inflammation [27], may hold promise as an adjuvant agent, but it does not appear to be sufficient as a standalone agent.

Cooling air devices for post‐procedure care were shown to be ineffective in preventing PIH in our analysis. Although conservative approaches previously recommended generous cooling, perhaps alternative strategies warrant further consideration. For example, adjusting the pulse duration and intervals between treatments could be considered [17].

Lastly, combinatorial measures do not have substantial sample sizes to appreciate conclusive outcomes, and further research is warranted. Studies of other preoperative treatments, for example, hydroquinone and vitamin C, have not been replicated on SOC patients yet [9].

This study has several limitations. First, the absence of a standardised scale for assessing PIH, with unstandardised photography approaches, poses a challenge for consistent evaluation. To mitigate these challenges, a mixed methodology was employed to capture the outcomes to the best of our ability. Although this review managed to focus on all SOCs, the results failed to capture other ethnicities/races. In addition, comparison with non‐SOC individuals was precluded by our exclusion criteria. While our review identified trauma‐induced PIH, PIH can originate from both internal and external factors, and this is a notable gap in the existing literature. Furthermore, the small sample sizes for many treatments and the inconsistency in reporting variables further contribute to the study's limitations.

## 5 Conclusion

While our study focused on darker skin tones, a distinct gap persists in incorporating all darker skin tones and ethnicities in research. There is also a lack of standardisation in outcome assessment that emphasises the necessity for the development of a universal PIH scale. While photoprotection is a promising preventative strategy, an educational gap exists wherein individuals with darker skin tones lack awareness regarding the significance of sunscreen usage. Many other interventions are far less effective and have only been studied on scarce sample sizes. In summary, there is substantial room for improvement in this area, and there is considerable optimism for enhancing preventative strategies in the future.

## Author Contributions

Authors made significant contribution to study conception and design, data collection, analysis and interpretation of results, and draft manuscript preparation. All authors read and approved the final version.

## Ethics Statement

The authors have nothing to report.

## Consent

The authors have nothing to report.

## Conflicts of Interest

The authors declare no conflicts of interest.

## Supporting information


Figure S1.

Table S1.

## Data Availability

The authors have nothing to report.
