# Supplementary material for: Prevention of Post‐Inflammatory Hyperpigmentation in Skin of Colour: A Systematic Review
Source: Australas J Dermatol. 2025 Feb 14;66(3):119–26. doi: 10.1111/ajd.14432 (PMC12062726; doi:10.1111/ajd.14432)
Supplement: Supplementary file 1 — Figure S1. [file AJD-66-119-s001.pdf]

Supplementary Figure 1. Summary of patient characteristics and prevention outcomes											
**Author, Year, Country**	**Sample size (n), Sex (M:F)**	**Mean Age, (Age range)**	**Race / Ethnicity (n), Fitzpatrick**	**Morphology (n) / Location (n)**	**Duration of PIH (days)**	**Precipitating Factor (n)**	**Treatment(s) / Prevention(s)**	**Outcomes**	**Follow up in months (n)**	**Recurrence**	**Adverse Events**
Lindgren et al., 2021, USA	1 (0:1)	20	NR , IV (1)	NR / face	NR	Acne (1)	Sunscreen, chemical peels, oral TXA, topical retinoid, spironolcatone (1)	Prevented (1)	NR	NR	None
Puaratanaarunkon & Asawanonda, 2022, Thailand	59 (17:42)	29 (19-46)	NR, III (33) IV (26)	NR, Face (59)	NR	Acne	Sunscreen and anti-inflammatory agents (59)	Prevented (59)	1.5 (59)	NR	Burning (7), redness (4), dryness (2), itching (1)
							Sunscreen (59)	Prevented (58), failed to prevent (1)	1.5 (59)	NR	NR
Bonciani & Cannarozzo, 2021, Italy	6 (0:6)	NR	NR, III (1) IV (5)	NR / Face (6)	NR	Solar lentigines (6)	Topical kojic acid, other (7)	Prevented (6), failed to prevent (1)	1 (7)	NR	NR
							Suncreen (7)	Prevented (4), failed to prevent (3)	1 (7)	NR	NR
Hong et al., 2018, Korea	1 (1:0)	58	NR / IV (1)	Macule / Face (1)	NR	Solar lentigines (1)	Topical α-agonists (1)	Prevented (1)	6 (1)	0	NR
							Topical steroids (1)	Failed to prevent (1)	6 (1)	0	NR
Techapichetvanich et al., 2018, Thailand	19 (3:16)	31.2 (18-60)	NR / III (5) IV (13) V (1)	NR / Face (19)	NR	Laser (19)	Topical EGF cream (19)	Prevented (9), failed to prevent (10)	2 (19)	NR	Stinging sensation (6), pustular (1), acneform reaction (2), melasma (1)
							Control (19)	Prevented (8), failed to prevent (11)	2 (19)	NR	Stinging sensation (4)
Vachiramon et al., 2018, Thailand	20 (8:12)	31.5 (26-38)	NR / III (14) IV (6)	NR	NR	IPL (20)	High-intensity focused ultrasound (20)	Prevented (8)	1 (20)	NR	Erythema (20), edema (20), skin tenderness (20), linear striations (NR), erosions (NR), ecchymosis (1)
							Control (20)	Prevented (12)	1 (20)	NR	NR

Supplementary Figure 1. Summary of patient characteristics and prevention outcomes											
**Author, Year, Country**	**Sample size (n), Sex (M:F)**	**Mean Age, (Age range)**	**Race / Ethnicity (n), Fitzpatrick**	**Morphology (n) / Location (n)**	**Duration of PIH (days)**	**Precipitating Factor (n)**	**Treatment(s) / Prevention(s)**	**Outcomes**	**Follow up in months (n)**	**Recurrence**	**Adverse Events**
Park et al., 2015, Korea	13 (2:11)	56.9 (41-69)	Asian (13) / III (3) IV (10)	NR	180	Solar lentignes (13)	Topical epidermal growth factor cream (13)	Prevented (12), failed to prevent (1)	1 (13)	NR	NR
	12 (2:10)	54.3 (40-64)	Asian (13) / III (5) IV (7)	Macule (25) / NR	NR	Solar lentignes (12)	Control (12)	Prevented (6), failed to prevent (6)	1 (12)	NR	NR
Nutjira Cheyasa et al., 2015, Thailand	40 (19:21)	NR	NR / IV (40)	NR / Face (40)	180	Laser (40)	Topical clobetasol (40)	Prevented (24), failed to prevent (16)	3 (40)	NR	Acneiform eruption (2)
							Control (40)	Prevented (10), failed to prevent (30)	3 (40)	NR	Acneiform eruption (1)
Wanitphakdeedecha et al., 2014, Thailand	26 (2:24)	34 (20-65)	Asian (26) / NR	NR	NR	Laser (26)	Sunscreen and anti-inflammatory agents (26)	Prevented (26)	3 (26)	NR	NR
Manuskiatti et al., 2007, Thailand	21 (0:21)	43 (27-72)	Asian (21) / III (16) IV (5)	Light brown macule (21) / Face (21)	NR	Laser (21)	Cooling air device (21)	Failed to prevent (21)	3 (21)	NR	Transient increased PIH (NR), non-resolved PIH (1)
Wei et al., 2019, China	30 (16:14)	26 (20-32)	Asian (30) / III (9) IV (18) V	Face (30)	NR	Laser (30)	Topical fusidic acid cream (29)	Failed to prevent (29)	4 (59)	NR	NR
	30 (15:15)	25 (19-31)	Asian (30) / III (9) IV (17) V (4)	Face (30)	NR	Laser (30)	Topical erythromycin eye ointment (30)	Failed to prevent (30)	NR	NR	NR
Sirithanabadeekul & Srieakpanit,	25 (7:18)	61 (NR)	NR / III (5) IV (17) V (3)	Macules / Extremities (25)	NR	Solar lentigines (25)	Intradermal TXA agent (25)	Reduced pigment (25)	3 (25)	NR	Burning (2)
							Control (25)	No reponse (1)	3 (25)	NR	NR

HEPES: 4-(2-hydroxyethyl)-1-piperazineethanesulfonic acid; IPL: intense pulsed light; LHA: Lipo Hydroxy Acid; TXA: tranexamic acid; EGF: Epidermal growth factor; QSNY: Q-switched neodymium-doped yttrium aluminum garnet; CO2: Carbon dioxide; SA: Salicylic acid; GA: Glycolic acid; JS: Jessner's solution; NR: Not reported
