# Supplementary material for: Prevention of Post‐Inflammatory Hyperpigmentation in Skin of Colour: A Systematic Review
Source: Australas J Dermatol. 2025 Feb 14;66(3):119–26. doi: 10.1111/ajd.14432 (PMC12062726; doi:10.1111/ajd.14432)
Supplement: Supplementary file 2 — Table S1. [file AJD-66-119-s002.docx]

**Supplementary Table 1.** Included studies

| **Country (%)** | Thailand | 57.1 |
| --- | --- | --- |
|  | Korea | 14.3 |
|  | United States | 7.1 |
|  | Italy | 7.1 |
|  | Japan | 7.1 |
|  | China | 7.1 |
|  | **Total** | 14 |
| **Study type (%)** | RCT | 57.1 |
|  | Experimental | 21.4 |
|  | Case report | 14.3 |
|  | Case series | 7.1 |
|  | **Total** | 14 |
| RCT: Randomized controlled trial; Non-RCT: Non-randomized controlled trial | | |
